# Upregulation of PCED1B-AS1 in proliferative diabetic retinopathy and its involvement in retinal vascular endothelial cell proliferation

**DOI:** 10.1186/s12886-022-02683-6

**Published:** 2022-11-23

**Authors:** Xuyang Wang, Wangling Chen, Wei Lao, Yunxin Chen

**Affiliations:** Hainan Eye Hospital and Key Laboratory of Ophthalmology, Zhongshan Ophthalmic Center, Sun Yat-sen University, 19 Xiuhua Road, 570311 Haikou, Hainan China

**Keywords:** PCED1B-AS1, Proliferative diabetic retinopathy, Biomarker, Anti-proliferation

## Abstract

**Background:**

This study was to assess the diagnostic value of PCED1B-AS1 for proliferative diabetic retinopathy (PDR) and investigate the involvement of PCED1B-AS1 in PDR.

**Methods:**

The vitreous and blood specimens from 37 subjects with PDR and 21 non-diabetics were examined by reverse transcription quantitative PCR to determine the PCED1B-AS1 level. The two groups were age- and gender-matched. Receiver operating characteristic (ROC) curves were plotted to visually illustrate the diagnostic ability of PCED1B-AS1. Human retinal Müller glial cells were studied by ELISA. Proliferation and migration of human retinal microvascular endothelial cells (HRMECs) were assessed in vitro.

**Results:**

Significant increases of PCED1B-AS1 levels were observed in the vitreous samples and CD34 + VEGFR-2 + cells from blood samples of diabetic subjects with PDR, compared with those of non-diabetics. The ROC curve based on the vitreous PCED1B-AS1 levels revealed an AUC of 0.812, while the ROC curve based on the PCED1B-AS1 levels in CD34 + VEGFR-2 + cells from blood samples revealed an AUC of 0.870. In Müller cell cultures, PCED1B-AS1 siRNA significantly attenuated VEGF and MCP-1 upregulation which were induced by CoCl_2_ and TNF-α. Additionally, PCED1B-AS1 siRNA attenuated VEGF-induced proliferation and migration in HRMECs.

**Conclusion:**

This study revealed the potential of PCED1B-AS1 as a diagnostic biomarker for PDR. In vitro data point to the anti-angiogenic and anti-proliferation effects of PCED1B-AS1.

## Background

Diabetic retinopathy (DR) is retinal vasculitis caused by diabetes mellitus manifesting as ophthalmological changes of neovascularization and macular edema [1, 2]. Initial DR is characterized by typical vascular and retinal abnormalities. The progression toward the stage of proliferative diabetic retinopathy (PDR) includes the development of persistent/chronic macular edema and marked fibrovascular proliferation [3]. Anti-vascular endothelial growth factor (VEGF) agents are widely used to block the progress from DME to PDR [6]. Increasing research support that genetic heritability would play an essential role in accounting for the susceptibility and development of PDR [7]. In this regard, heritability can estimate this late diabetic complication with 25–50% accuracy [8, 9]. Molecular alterations of the vitreous during diabetic eye disease can reflect the metabolic and functional modifications of the vitreoretinal interface caused by pathological conditions. In PDR, the vitreum represents a “reservoir” of pathological signaling molecules. Therefore, the vitreum from PDR patients can be a novel tool to obtain experimental evidence of new therapeutic targets The structural and molecular changes can reflect pathological events occurring at the vitreous body retina interface and can characterize the condition of diabetes [12]. In turn, the vitreous body changes have a pathological effect on the diabetic retina, which leads to a vicious spiral and disease progression. In PDR, the vitreous body is characterized by significant changes in its molecular composition, including long non-coding RNAs (lncRNAs) [13]. Therefore, investigating lncRNA changes occurring in non-diabetic versus diabetic vitreous may provide useful insights into the development and pathological processes during PDR.

Non-coding RNAs are a group of bioactive molecules that can be obtained from the vitreous of patients with PDR by pars plana vitrectomy [14]. LncRNAsare non-coding RNAs that are longer than 200 nucleotides. LncRNAs are capable of regulation in various physiological and pathological processes, such as angiogenesis and inflammation [15]. Increasing studies have made it evident that PDR is characterized by marked changes in lncRNA expression [16]. A study by Liu et al. screened 82 dysregulated lncRNAs by high-throughput sequencing from PDR patients and confirmed using reverse transcription quantitative PCR [17]. Moreover, a study examining the samples of the human vitreous from proliferative DR found linc00174 was significantly elevated in PDR patients, and this lncRNA deteriorates diabetic retinal microangiopathy via regulating VEGF pathway [18]. The possible involvement of lncRNA dysregulation opens a new horizon of research in PDR. PCED1B Antisense RNA 1 (PCED1B-AS1) was revealed to contribute to the modification of tumor immune microenvironment, promote the proliferation of colorectal adenocarcinoma, and regulate terminal erythroid differentiation [19–21]. PCED1B-AS1 (AL024507.2) has been screened as a DR-associated lncRNA in a previous study [22]. But the further dissection of its significance in PDR is lacking.

This study aimed to determine the alteration of PCED1B-AS1 that occurs in the vitreous and blood of patients with PDR, to explore the role of PCED1B-AS1 in the development of PDR, and to assess the diagnostic potential for PDR.

## Methods

### Patient selection

Eligible subjects had type 1 or 2 diabetes mellitus combined with PDR and were aged more than 18 years. Subjects with the presence of glaucoma or other retinopathy not related to diabetes mellitus were ineligible. This study collected 37 patients with PDR and 21 individuals without diabetes mellitus who were scheduled for vitrectomy surgery at Zhongshan Opthalmic Center, Sun Yat-sen University from March 2017 to March 2019. The clinical information of these participants was collected from medical records in our institution. The patients in two groups were assigned random digit codes and presented to assessors using randomized presentation orders. All the diagnoses were separately made by retinal specialists who were masked to the groups. PDR was diagnosedbased on the clinical symptoms and clinical imaging according to the International Clinical Diabetic Retinopathy Disease Severity Scale. The 21 nondiabetic patients with macular holes (*n* = 12) or idiopathic macular epiretinal membrane (*n* = 9) according to International Clinical Diabetic Macular Edema Disease Severity Scale were enrolled as the control group. The intraclass correlation analysis (ICC) ranged from 0.88 to 0.94, indicating high agreement between assessors.

All procedures performed in studies involving human participants were in accordance with the ethical standards of the institutional and/or national research committee and with the 1964 Helsinki Declaration and its later amendments or comparable ethical standards. The study was approved by the Research Centre and Institutional Review Board of Zhongshan Opthalmic Center, Sun Yat-sen University. All patients gave preoperative informed written consent and approved the use of the vitreous fluid for further research.

### Human vitreous samples preparation

A standard vitrectomy surgery was performed at the Alcon Constellation Systems (Alcon Nordic, USA). Approximately 1 mL of undiluted vitreous sample was collected. The undiluted vitreous samples were kept at 4 ℃ and promptly transported for centrifugation (10,000 g, 10 min). Then, the supernatants were aliquoted and placed in a liquid nitrogen tank until assay.

### Human peripheral blood samples preparation

After overnight fasting, 5 mL peripheral blood samples were collected in BD Vacutainer® CPT™ Tubes (USA) from the subjects. The tubes were gently mixed upside down 10 times and stored vertically. Within two hours, the tubes were centrifugated at 1500 g, 20 min, and 25 °C. The mononuclear precursor cells were isolated, washed, and resuspended in PBS (Gibco, USA). The isolated cells were incubated with two kinds of monoclonal antibodies: PE-conjugated anti-human CD34 (BD, USA) and allophycocyanin-conjugated anti-human VEGFR-2 (R&D Systems, USA). The cell sorting was performed on a FACSAria III (BD Biosciences, USA) at 488 nm to obtain double-positive cells, CD34 + VEGFR-2 + cells [23].

### PCED1B-AS1 siRNA

SiRNA specifically against PCED1B-AS1 and siRNA negative control (si-Ctrl) were provided by Biomics Biotechnologies (Jiangsu, China). These plasmids were transfected into cells using Lipofectamine™ 3000 Reagent abiding by the manufacturer’s instruction (Invitrogen, USA).

### Human retinal Müller glial cell culture

The human Müller cell line was obtained from ScienCell (USA), and grown in high glucose DMEM (Gibco, USA) containing 10% fetal bovine serum (Gibco, Australia). The 26th passage of Müller cells were incubated at 37 °C in 5% CO_2_ until monolayer confluence. Then, the cells were either left without transfection or transfected with indicated plasmids for 24 h. The transfected cells were subjected to the following stimuli, respectively: 25 mM glucose (Sigma-Aldrich, USA), 300 µM of the hypoxia mimetic agent cobalt chloride (CoCl_2_) (Sigma-Aldrich, USA), or 50 ng/mL TNF-α human (Sigma-Aldrich, USA) [24]. For high-glucose treatment, 25 mM mannitol (Sigma-Aldrich, USA) was used as a control. Cell culture mediums were collected for Enzyme-Linked Immunosorbent Assay (ELISA).

### Human retinal microvascular endothelial cell (HRMECs) culture

HRMECs were subscribed from Cell Systems Corporation (USA) and grown in Endothelial Basal Medium-2 (Lonza, Switzerland) supplemented with SingleQuots kit (Lonza). HRMECs up to passage 8 were grown at about 80% confluency. After transfection, the cells were starved in a minimal medium overnight to eliminate the effects of growth factors. Then cells were stimulated by 10 ng/mL Recombinant Human VEGF_165_(R&D Systems, USA).

### Reverse transcription quantitative PCR

Total RNA, including lncRNAs, was extracted from the harvested cells or processed vitreous samples using TriZol reagent (Life technology, USA). The cDNA was prepared using RevertAid First Strand cDNA Synthesis Kit (Thermo Fisher, USA) as per the manufacturer’s instruction. Reverse transcription quantitative PCR was performed using SYBR Green qPCR Master Mix (High ROX) (MedChemExpress, USA) at an ABI PRISM 7000 (Applied Biosystems, USA). The primers used were as follows: 5ʹ-TTTTGATGTTGGCCAATGCCG-3ʹ and 5ʹ-GGGCAGGGAGTCTTCATAGC-3ʹ for PCED1B-AS1; 5ʹ-AGAGGCAGGGATGATGTTCTG-3ʹ and 5ʹ-GACTCATGACCACAGTCCATGC-3ʹ for glyceraldehyde-3-phosphate dehydrogenase (GAPDH). The relative expression of PCED1B-AS1 was calculated using the 2^−∆∆Ct^ method with GAPDH as an endogenous control.

### ELISA assays

Human MCP1 ELISA Kit (1.56pg/mL) and Human VEGF ELISA Kit (< 1pg/mL) were both purchased from Biorbyt (United Kingdom). Determination of human chemotactic protein-1 (MCP-1) and VEGF in culture medium was carried out with the aforementioned ELISA kits according to the assay protocol notes in the respective manual.

### HRMECs proliferation assay

To determine whether PCED1B-AS1 influences HRMECs proliferation, cell viability was measured by the nonradioactive CellTiter 96® aqueous one solution (Promega, USA). First, approximately 3,500 transfected or non-transfected cells were added to each well of 96-well plates at 37 °C for 24 h. After 24 h, HRMECs were starved overnight. Then HRMECs were incubated with or without 10 ng/mL of 10 ng/mL Recombinant Human VEGF_165_ (R&D Systems, USA). After 24 h of incubation. 20 µL of CellTiter 96® AQueous One Solution was transferred to each well following another 3 h of incubation at 37 °C. The absorbance at 490 nm was read at a microplate reader (Bio-Rad, USA) to represent the number of living cells.

### HRMECs migration assay

The HRMECs migration assay was performed using Boyden24-well Transwell plates and chambers (Corning Costar, USA). Briefly, the lower surface of the filter was coated with 2% gelatin (10 µg). Endothelial Basal Medium-2 (1% FBS) containing 10 ng/mL VEGF-A (Abcam, USA) was placed in the lower chamber. Transfected or non-transfected HRMECs (1 × 10^5^ cells/mL of medium containing 1% FBS) were loaded into each of the upper wells. After incubation for 4 h in a CO_2_ incubator, the cells were fixed and stained with crystal violet. Chemotaxis was quantified using a computer-aided inverted phase-contrast microscope after the elimination of the non-migrating cells in the upper surface of the filter.

### Statistical analysis

The shapiro-Wilk test was used to assess the normality of data. A Chi-square test was carried out to compare the age of patients between PDR and controls. Independent samples t-test was used to compare the other clinical data between patients with PDR and controls. The data between the two groups were analyzed by Student t test. The correlation between the expression of PCED1B-AS1 in human vitreous and blood samples was determined using Pearson’s test and linear regression. Significance was denoted at *P* < 0.05.

## Results

### The clinical characteristics of the study population

**Fig. 1:**
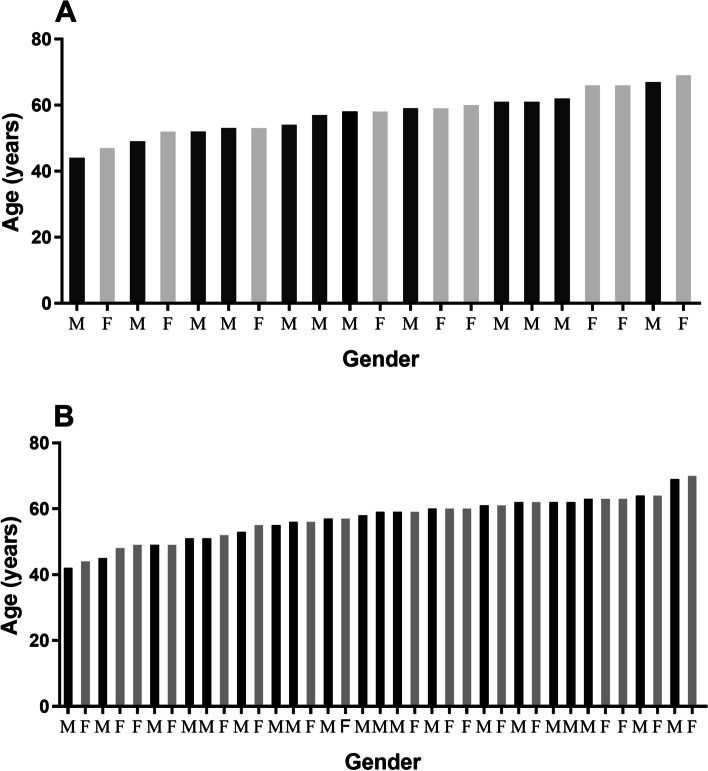
Age and gender distribution of the study population. **A **Age and gender distribution of the non-diabetic subjects. **B** Age and gender distribution of patients with PDR. M, male; F, female

Thirty-seven patients with PDR and twenty-one individuals without diabetes were recruited in this study. Individuals from these two groups were age-matched (57.47 ± 6.71 years vs. 57.03 ± 6.72 years), and gender-matched (female/male: 17/20 males vs. 9/12; Fig. 1). There was no difference in the levels of body mass index, total cholesterol, and triglyceride in the PDR group and the non-diabetic group (Table 1). In the PDR group, two patients had neuropathy complications. Regarding additional therapies, 16 patients received insulin treatment, 9 received antihypertension treatment, and 6 received photocoagulation treatment.

**Table 1:** Clinical characteristics of the study population

Paraments	PDR (*n* = 37)	Control (*n* = 21)	*P* value
Age (years)^a^	57.03 ± 6.72	57.48 ± 6.71	0.808
Male (female/male), n^b^	17/20	9/12	0.820
Body mass index (kg/m^2^)^a^	22.78 ± 2.41	22.12 ± 1.94	0.290
HbA1c (%)^a^	6.74 ± 0.85	4.83 ± 0.44	< 0.001
Total cholesterol (mmol/L)^a^	4.66 ± 0.53	4.39 ± 0.69	0.097
Triglyceride (mmol/L)^a^	1.57 ± 0.45	1.46 ± 0.46	0.371
Other diabetic complications, n	2/34	0/21	n.a.
Treatments, n			
Insulin treatment	16	n.a.	n.a.
Antihypertension	9	n.a.	n.a.
Photocoagulation	6	n.a.	n.a.

n.a., not applicable

^a^Independent samples

^b^Chi-square test

### Upregulated PCED1B-AS1 in human vitreous and blood samples

**Fig. 2:**
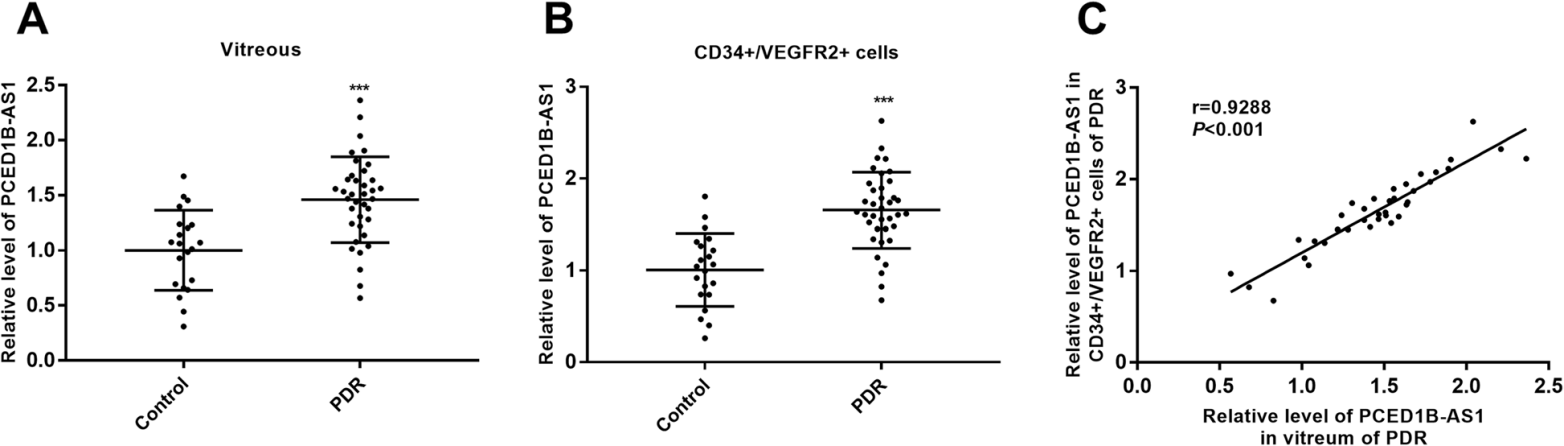
Altered levels of PCED1B-AS1. **A** Upregulated level of PCED1B-AS1 in the vitreous humor of non-diabetic patients and PDR patients was determined by reverse transcription quantitative PCR. ***P < 0.001 (Unpaired -t-test). **B** Upregulated levels of PCED1B-AS1 in the CD34 + VEGFR-2 + cells from blood samples were quantified by reverse transcription quantitative PCR assay. ***P < 0.001 (Unpaired t-test). **C** The level of PCED1B-AS1 in the vitreous humor and the CD34 + VEGFR-2 + cells from blood samples of non-diabetic patients and PDR patients showed a linear correlation. ***P < 0.001 (Pearson r: 0.9288)

Assessment of PCED1B-AS1 in the vitreous and the CD34 + VEGFR-2 + cells from blood samples was archived by reverse transcription quantitative PCR assay. The results showed a significant increase of PCED1B-AS1 levels in the vitreous samples of diabetic subjects with PDR compared with those of the control group without diabetes (*P* < 0.001; Fig. 2 A). Additionally, the CD34 + VEGFR-2 + cells from blood samples of patients with PDR represented a markedly increased level compared to nondiabetic subjects (*P* < 0.001; Fig. 2B). Of note, the levels of PCED1B-AS1 in human vitreous and blood samples were positively correlated after Pearson’s test and linear regression (r = 0.9288, *P* < 0.001; Fig. 2 C). These results indicated that PCED1B-AS1 was upregulated in both human vitreous and blood samples.

### Diagnostic accuracy of PCED1B-AS1

**Fig. 3:**
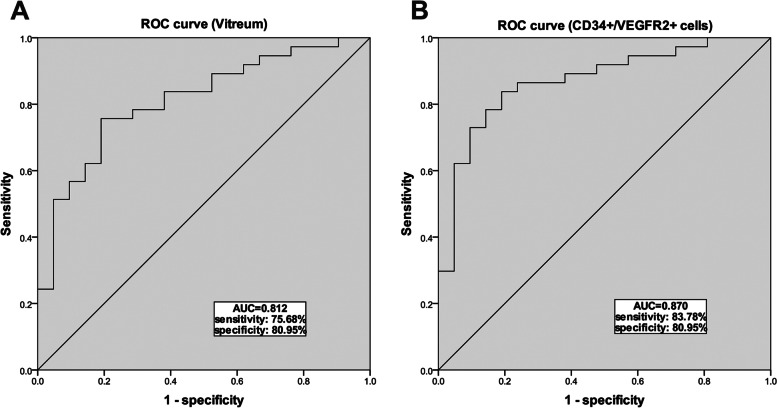
Receiver operating characteristic (ROC) curves were plotted to determine the diagnostic ability of PCED1B-AS1 for PDR. **A** ROC curve of PCED1B-AS1 based on the expression in vitreous of non-diabetic patients and PDR patients. **B** ROC curve of PCED1B-AS1 based on the expression in the CD34 + VEGFR-2 + cells from blood samples of non-diabetic patients and PDR patients

The ROC curves and AUC values for PCED1B-AS1 in vitreous and blood samples are shown in Fig. 3. As shown, the PCED1B-AS1 in vitreous provided high diagnostic information with an AUC of 0.812 (Sensitivity: 75.68%, specificity: 80.95%; Fig. 3 A). The ROC curve of the PCED1B-AS1 in CD34 + VEGFR-2 + cells from blood samples had an AUC of 0.870 (Sensitivity: 83.78%, specificity: 80.95%; Fig. 3B).

### Downregulated PCED1B-AS1 attenuated the levels of angiogenic and inflammatory molecules in human retinal Müller glial cells.

**Fig. 4:**
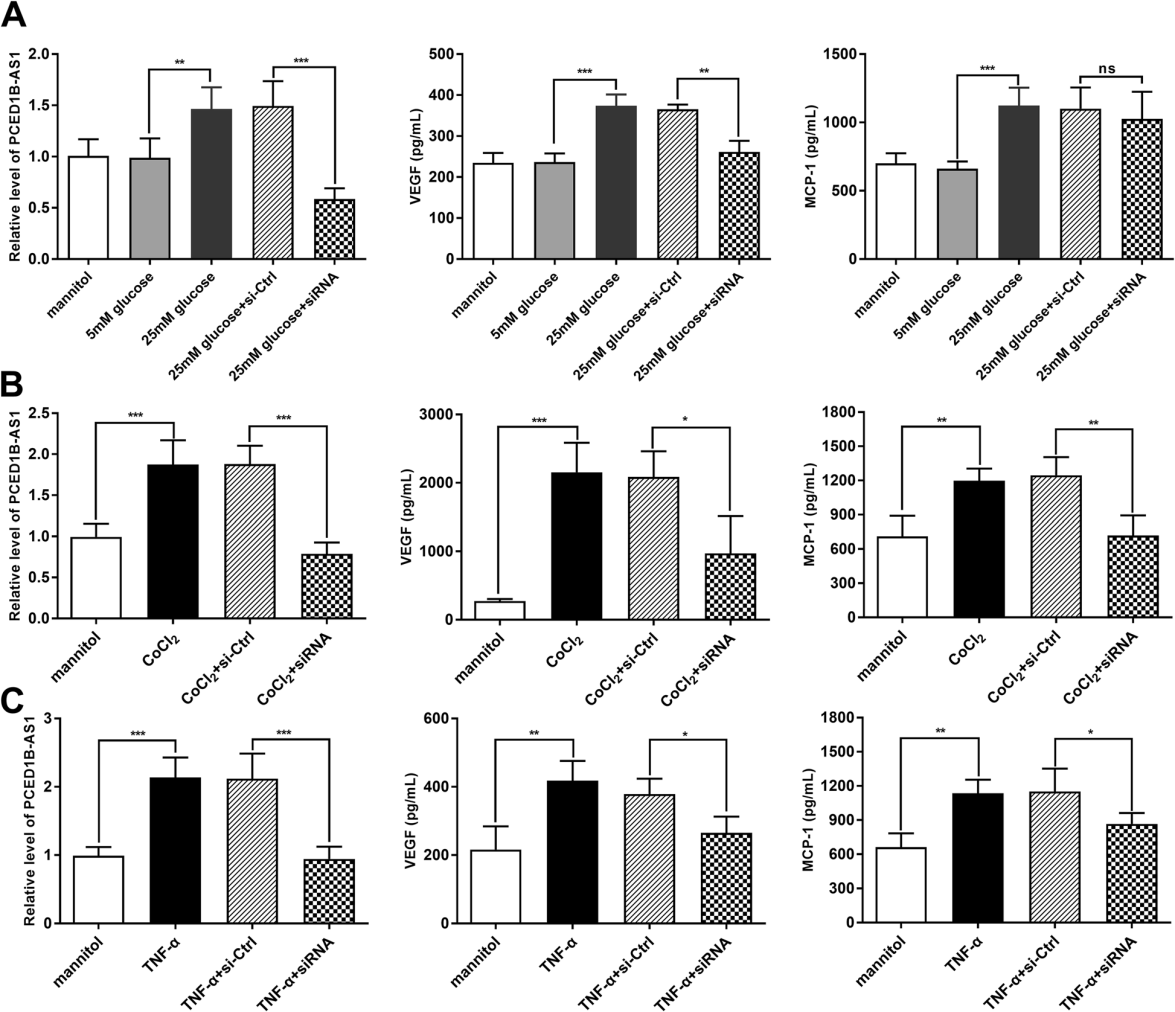
Downregulated PCED1B-AS1 affected the levels of angiogenic and inflammatory molecules in human retinal Müller glial cells. **A** Human retinal Müller glial cells with PCED1B-AS1 siRNA-transfection or without were left untreated or treated with 25 mM glucose, levels of VEGF and MCP-1 in the culture media were quantified by ELISA. **B** Human retinal Müller glial cells with PCED1B-AS1 siRNA-transfection or without were left untreated or treated with 300 µM CoCl_2_, and levels of VEGF and MCP-1 in the culture media were quantified by ELISA. **C** Human retinal Müller glial cells with PCED1B-AS1 siRNA-transfection or without were left untreated or treated with 50 ng/ml TNF-α, and levels of VEGF and MCP-1 in the culture media were quantified by ELISA. *P < 0.05, **P < 0.001, ***P < 0.001 (Unpaired t-test)

To better understand the effect of PCED1B-AS1 on PDR, we investigated the molecular effects of PCED1B-AS1 on human retinal Müller glial cells using various experimental conditions associated with DR. Through ELISA analysis, the results demonstrated that the stimulation of Müller cells with the diabetic mimetic conditions 25 mM glucose (Fig. 4 A), 300 µM of the hypoxia mimetic agent CoCl_2_ (Fig. 4B), or the proinflammatory cytokine 50 ng/ml TNF-α human (Fig. 4 C) induced the increase of PCED1B-AS1 levels to different degrees (*P* < 0.01). Significant upregulations were observed in levels of VEGF and MCP-1 in the culture medium containing 25 mM glucose as compared to respective controls. Müller cells with pre-transfection of PCED1B-AS1 siRNA significantly attenuated the levels of VEGF induced by 25 mM glucose, CoCl_2_, and TNF-α. In addition, PCED1B-AS1 siRNA significantly attenuated upregulation of MCP-1 induced by CoCl_2_ and TNF-α.

### Downregulated PCED1B-AS1 reduced the VEGF-induced proliferation and migration of HRMECs

**Fig. 5:**
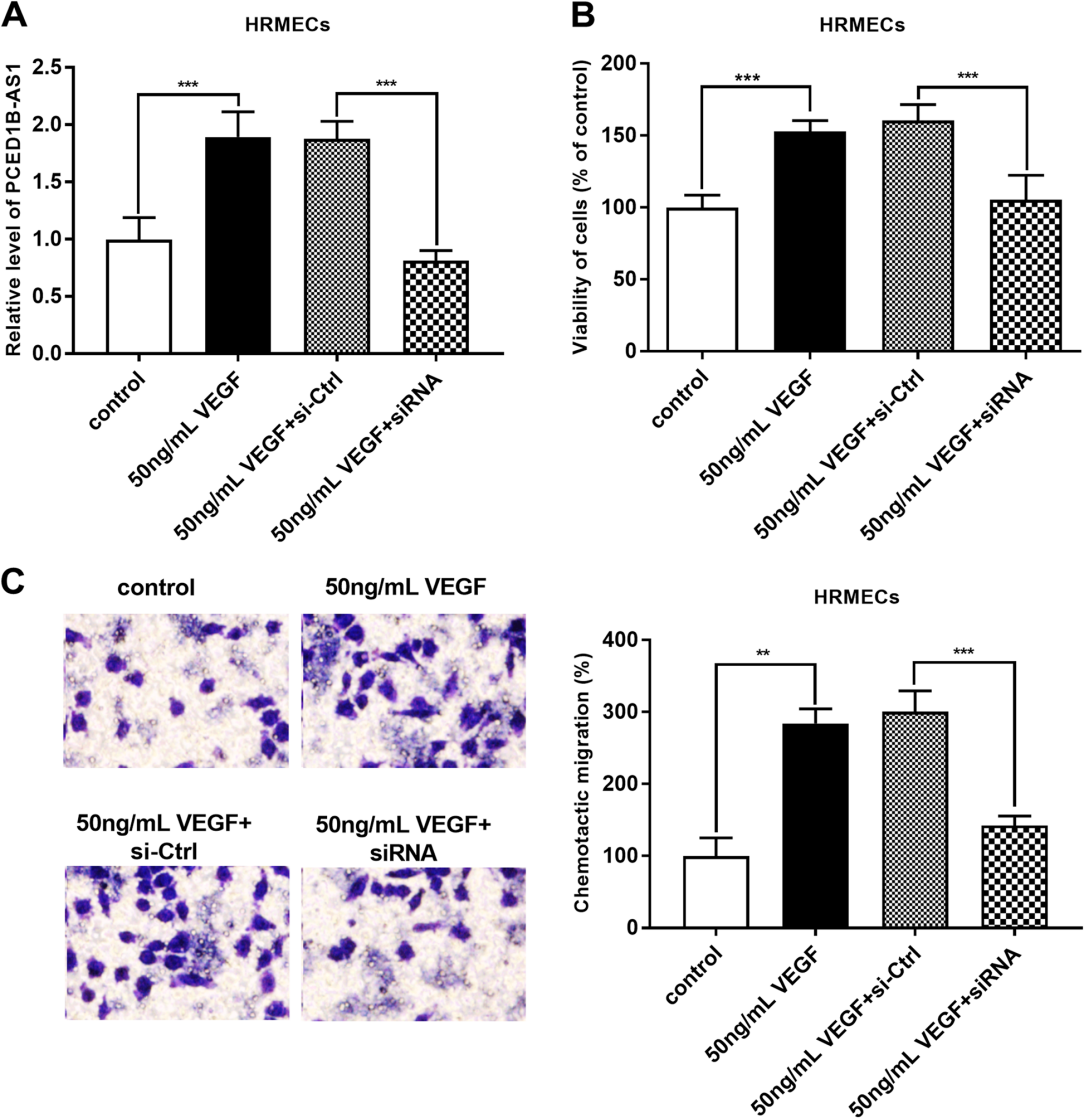
Downregulated PCED1B-AS1 affected the proliferation and migration of HRVECs. **A** VEGF led to an increased level of PCED1B-AS1 in HRVECs. **B** Downregulated PCED1B-AS1 inhibited HRVECs survival induced by VEGF. **C** Downregulated PCED1B-AS1 inhibited HRVECs migration induced by VEGF. **P < 0.01, ***P < 0.001 (Unpaired t-test)

To determine the effect of PCED1B-AS1 on angiogenesis in vitro, HRMECs were incubated with VEGF and the proliferation assay and migration assay were monitored. The VEGF treatment can increase the expression level of PCED1B-AS1, but this increase can be brought down by PCED1B-AS1 siRNA as shown in Fig. 5A (*P* < 0.001). VEGF treatment stimulated the proliferation of HRMECs, conversely, PCED1B-AS1 siRNA suppressed the VEGF-induced proliferation of HRMECs (*P* < 0.01, Fig. 5B). With the treatment of VEGF, the migratory ability of HRMECs was increased, which was significantly weakened by PCED1B-AS1 siRNA (*P* < 0.01, Fig. 5C). Thus, PCED1B-AS1 siRNA can reduce the VEGF-induced proliferation and migration of HRMECs in vitro.

## Discussion

This study demonstrates the upregulation of PCED1B-AS1 in the retina in PDR and the subsequent effect on cell function. We have shown that the levels of PCED1B-AS1 in human vitreous and blood samples can be potential biomarkers to distinguish patients with PDR and individuals without diabetes. Therefore, PCED1B-AS1 has the potential to develop as a diagnostic molecular forPDR.

Traditionally, optical coherence tomography (OCT) help physician around the world to proper diagnosis not only for DME but also for varying grades of DR [25, 26]. Recently, the expression of some lncRNAs is abnormal in many diseases, and DR is no exception [27]. This gave birth to a potential application for lncRNAs as biomarkers for DR. For instance, lncRNA BANCR, MALAT1, and MIAT were previously described as upregulated lncRNAs in DR patients and have diagnostic potentials for DR [28–30]. Here, to compare the circulating level and vitreous level of the lncRNA in PDR patients, we collected the blood samples and vitreous bodies from both PDR and non-diabetic individuals. We then showed that PCED1B-AS1 was strongly upregulated in the retinas of PDR patients. CD34+/VEGFR2 + mononuclear cells were identified as circulating endothelial progenitor cells (EPCs) [31]. EPCs carry monocyte markers for early retinopathy [23]. The expression of PCED1B-AS1 in CD34+/VEGFR2 + cells from blood confirmed the upregulated status of PCED1B-AS1 in PDR patients. Consistently, high glucose conditions trigger the upregulation of PCED1B-AS1 in vitro. The upregulation of PCED1B-AS1 in Zhu et al. [22] study also supports our results and provided additional evidence of PCED1B-AS1 in PDR from a bioinformatics perspective. Then, after ROC analysis, PECD1B-AS1 showed a strong ability to distinguish PDR from the non-diabetic. Bischoff et al. reported that lncRNAs detected in peripheral blood might be suitable as biomarkers for disease [32]. Thus, circulating and vitreous PCED1B-AS1 was proven very powerful as a biomarker for PDR.

PDR is characterized by the neovascularization of various types of hemorrhage and pathological proliferation of retinal vessels.[25, 26] LncRNAs can affect the development of non-proliferative DR to PDR by influencing inflammation, angiogenesis, and retinal cell proliferation [33]. DR was initially considered to be a microvascular complication of diabetes. However, there is increasing evidence that neurodegeneration is an early event in its pathogenesis [34]. In fact, an abnormal retinal function is also detected in patients without any microvascular abnormalities. Therefore, the American Diabetes Association (ADA) recently defined DR as a highly specific neurovascular complication [35]. With the use of Human retinal Müller glial cells and HRVECs in vitro studies, we tried to obtain insights into the mechanism of PCED1B-AS1 in PDR. In this study, we confirmed that high glucose, hypoxia mimetic agent CoCl_2_, or the proinflammatory cytokine TNF-a induced a clear upregulation of PCED1B-AS1 in Müller cells. The retinal expression of Müller cells is recognized as the main secretion source of VEGF in the retina [36]. In these three types of simulation conditions, the level of VEGF was increased. However, we demonstrated that PCED1B-AS1 downregulation attenuated the upregulation of VEGF in human retinal Müller glial cells induced by hypoxia-mimetic, high-glucose, or proinflammatory-mimetic conditions. MCP-1 has been stated as a chemokine that promotes the migration and recruitment of monocytes [37]. Additionally, this study observed that PCED1B-AS1 significantly attenuated the upregulation of MCP-1 induced by the CoCl_2_ or TNF-α in Müller cells. Our findings are in agreement with previous studies which demonstrated that PCED1B-AS1 modulates chemokine receptors and immunosuppression [38, 39]. The fact that PCED1B-AS1 had no effect on the upregulation of MCP-1 induced by the 25mM glucose may indicate that PCED1B-AS1 has a particular influence on the oxidative stress induced by continuous high glucose, instead of high glucose itself. Of course, this deduction should be further by a series of experiments including continuous normal glucose, continuous high glucose, alternating normal and high glucose, or mannitol. Angiogenesis is an essential element in the initiation and progression of PDR. VEGF plays a key role in promoting retinal vascular leakage and angiogenesis in PDR [40]. In addition, HRVECs in the high glucose circumstance will proliferate and migrate abnormally, leading to changes in retinal vascular function [19]. Accumulating evidence suggests that lncRNAs have important regulatory roles in high glucose-induced retinal vascular endothelial cell dysfunction [31]. After the initial identification of PCED1B-AS1 alteration in HRMECs induced by VEGF, the proangiogenic potential of PCED1B-AS1 was evaluated with the in vitro migration and proliferation assay. In the current study, PCED1B-AS1 inhibition was demonstrated to attenuate VEGF-induced HRMECs migration and proliferation. Similarly, several studies demonstrated that PCED1B-AS1 downregulation is a potent inhibitor of tumor-associated proliferation and migration [20, 41, 42]. Therefore, PCED1B-AS1 holds the potential for improved PDR treatment.

## Conclusions

In conclusion, this study demonstrated the important role of PCED1B-AS1 in PDR. In this study, we observed an upregulation of PCED1B-AS1 in PDR samples. And the levels of PCED1B-AS1 in vitreous and blood have the potential to distinguish PDR from non-diabetes. This study also demonstrated that ablation of PCED1B-AS1 had anti-inflammatory and anti-angiogenic effects in PDR. And this research could provide a new target for the clinical treatment of PDR. Although our research has provided some information on PCED1B-AS1 in PDR, we have not verified the mechanism of PCED1B-AS1 interacting with VEGF and exerting its biological effects. Understanding the mechanism of PCED1B-AS1 thoroughly can pave the way for the rational design of drugs that block the PDR.

## Data Availability

The datasets used and/or analysed during the current study are available from the corresponding author on reasonable request.

## Abbreviations

PDR: Proliferative diabetic retinopathy
ROC: Receiver operating characteristic
VEGF: Vascular endothelial growth factor
lncRNAs: Long non-coding RNAs
PCED1B-AS1: PCED1B Antisense RNA 1
ICC: Intraclass correlation analysis
CoCl2: Cobalt chloride
ELISA: Enzyme-Linked Immunosorbent Assay
HRMECs: Human retinal microvascular endothelial cell ()
GAPDH: Glyceraldehyde-3-phosphate dehydrogenase
OCT: Optical coherence tomography
EPCs: Endothelial progenitor cells
ADA: American Diabetes Association
